# Age and gender differences in health characteristics of older people with visual impairment

**DOI:** 10.1007/s40520-025-02998-6

**Published:** 2025-03-26

**Authors:** Salvatore Metanmo, Charnel Mbianda, Marie-Josiane Ntsama-Ebode, Callixte Kuate-Tegueu, Melanie Annick Magnerou, Nadine Simo, Antoine Gbessemehlan, Maturin Tabue-Teguo

**Affiliations:** 1 Department of Clinical Research and Innovation-CS 90632, University Hospitals of Martinique-Pierre Zobda-Quitman Hospital, Fort-de-France, 97261 Martinique; 2 Inserm UMR-S U1237, PhIND, BB@C, Caen, France; 3 Faculty of Medicine and Biomedical Sciences, University of Yaoundé I, Yaounde, Cameroon; 4 Université de Douala, Douala, Cameroon; 5 Centre Hospitalo-Universitaire de Martinique, Fort-de-France, France; 6 Inserm U1094, IRD Umr270, University Limoges, Epimact—Epidemiology of Chronic Diseases in Tropical Zone, Institute of Epidemiology and Tropical Neurology, Omegahealth, Limoges, France; 7 Department of Geriatrics, University Hospital of Martinique, Fort-de-France, Martinique

**Keywords:** Gender differences, Age differences, Visual impairment, Older people

## Abstract

**Background:**

Africa’s population is ageing, with an attendant increase in the number and duration of health problems such as visual impairment (VI). Few data are available from sub-Saharan Africa concerning age and gender differences in this population of people suffering from VI. The aim of this study was to examine age and gender differences in the health characteristics of older people with VI in Cameroon.

**Methods:**

This was a cross-sectional study carried out in Douala, Cameroon, among people aged 55 and over. Participants had to be free of blindness, neurological disorders or other severe health problems. Sociodemographic and clinical variables were collected, including VI. We investigated differences between men and women in different age strata (< 65, 65–75, > 75 years) among people with VI.

**Results:**

Among 403 participants, 356 (88.3%) had VI and, regardless of age group, there were significantly more women, who lived alone, were professionally active, overweight, had frailty syndrome and were less likely to drive a car. For other variables (education, chronic alcoholism, cognitive impairment, basic and instrumental activities of daily living, depression and falls), differences between men and women were only significant in certain age groups, with women generally at higher risk.

**Conclusion:**

The prevalence of VI is high older (> 55 years) adults in Cameroon, with clear differences between men and women. The oldest individuals, and women appear to be most vulnerable. The identification of at-risk groups/persons through this cross-sectional study should make it possible to adapt public health strategies to contribute to the WHO objective of healthy ageing.

## Introduction

The ageing of the world’s population is a significant demographic phenomenon. According to the World Health Organization (WHO), the proportion of the global population aged over 60 is projected to double by 2050, increasing from approximately 12–22% [1]. Low- and middle-income countries are experiencing, and will continue to experience the greatest changes in their age pyramids. By 2050, two-thirds of the world’s population aged over 60 will live in these countries. Between 2020 and 2050, the older African population is projected to triple, from 74.4 million to 235.1 million, and its growth in the older adult population in the next three decades will outpace that of any other region of the world [2]. This increase in the number of older people is positive news, insofar as it reflects longer lifespans. However, it is also associated with longer periods of living with functional decline, such as visual impairment (VI) [3].

In older adults, VI has been reported to be associated with limitations in the activities of daily living, reduced mobility, low social activities/connections [4–6], increased fall risk [7], adverse effects in terms of cognitive and mental health [8, 9], increased risk of chronic diseases, and reduced quality of life [6, 10].

The highest age-standardized prevalence of all forms of VI was found in older adults (aged 50 years and older) in Western Sub-Saharan Africa. Additionally, it has been widely reported that women generally bear the greatest burden of visual impairment, with age-standardized prevalence rates for all severities of VI being higher in females aged 50 years and older than in their male counterparts [11–13].

Although gender differences in VI are not systematic, studies generally report a higher prevalence of VI in women than in men. This difference is thought to be linked to factors such as exposure to risk factors, limited access to healthcare, and differences in treatment-seeking behaviour [14]; with women being generally more disadvantaged [15, 16]. One meta-analysis reported that severe VI due to cataracts could be reduced by around 11% in low- and middle-income countries if women underwent cataract surgery at the same rate as men [15].

With the exception of a few West African countries, such as Ghana, Gambia and Togo, which have carried out some work on gender inequalities/differences in the context of VI [17], there is little data available from sub-Saharan Africa, particularly from Cameroon. Yet, understanding these gender-based differences in VI, particularly among older adults in Sub-Saharan Africa, and their potential causes could help us to adapt healthcare systems to identify and better support disadvantaged groups. To fill this knowledge gap, this study aims to examine age- and sex-specific health characteristics of older individuals with VI in Douala, Cameroon.

## Methods

A cross-sectional population-based study was performed in the city of Douala in Cameroon between January 1 and March 31, 2019. Participants were members of MUPAC (a mutual health insurance company for older individuals in Cameroon). They were aged 55 years or older, and were able to stand and walk four meters unaided. Details of the survey procedures have previously been described elsewhere [18, 19]. We excluded blind participants and those with neurological disorders or other severe health problems. Numerous sociodemographic and clinical variables were collected, including self-reported VI.

### Statistical analysis

Participant characteristics are described according to the presence or absence of VI. Next, the statistical analysis focused on the population of those with VI, and describes the characteristics of the participants according to gender across three age categories, namely 55 to < 65, 65 to 75, and > 75 years. In each age group, differences between women and men were tested with the appropriate parametric or non-parametric statistical tests according to the distribution. *P*-values < 0.05 were considered statistically significant.

The study was approved by the Ethics Committee of the Université des Montagnes (Bangangté-Cameroon) under the number 2019/049/UdM/CIE. Written informed consent was obtained from all participants before inclusion in the study.

## Results

In total, 403 people were included in the study, median age was 67 years (interquartiles, 63–71), 49.6% were women and 356 (88.3%) reported having VI. Women, individuals with hypertension, those with a high depression score and those with frailty syndrome more frequently had VI. The distribution of the other characteristics was identical in both groups (Table 1).

**Table 1:** Distribution of characteristics according to the presence or absence of visual impairment

Characteristic	Overall N = 403^1^	No VI N = 47^1^	VI N = 356^1^	p-value^2^
Age				0.560
[55,65]	140 (34.7%)	18 (38.3%)	122 (34.3%)	
[65,75]	209 (51.9%)	25 (53.2%)	184 (51.7%)	
[75,88]	54 (13.4%)	4 (8.5%)	50 (14.0%)	
Age	67.0 (63.0, 71.0)	66.0 (62.5, 71.0)	67.0 (63.0, 71.3)	0.836
Sex (female)	200 (49.6%)	15 (31.9%)	185 (52.0%)	**0.010**
Live alone	176 (43.7%)	16 (34.0%)	160 (44.9%)	0.157
Scolarity (secondary or higher)	278 (69.0%)	31 (66.0%)	247 (69.4%)	0.633
Being professionally active	163 (40.4%)	22 (46.8%)	141 (39.6%)	0.344
Self-driving	80 (19.9%)	11 (23.4%)	69 (19.4%)	0.516
Overweight (body mass index ≥ 25)	287 (71.2%)	32 (68.1%)	255 (71.6%)	0.614
Diabetes	40 (9.9%)	3 (6.4%)	37 (10.4%)	0.602
Hypertension	121 (30.0%)	6 (12.8%)	115 (32.3%)	**0.006**
Stroke	9 (2.2%)	0 (0.0%)	9 (2.5%)	0.607
Chronic alcoholism	30 (7.4%)	4 (8.5%)	26 (7.3%)	0.767
Tobacco consumption	8 (2.0%)	2 (4.3%)	6 (1.7%)	0.237
Cognitive impairment	207 (51.4%)	22 (46.8%)	185 (52.0%)	0.506
ADL score	6.0 (6.0, 6.0)	6.0 (6.0, 6.0)	6.0 (6.0, 6.0)	0.372
IADL score	4.0 (4.0, 4.0)	4.0 (4.0, 4.0)	4.0 (4.0, 4.0)	0.589
Total CES-D	10.0 (8.0, 12.0)	10.0 (8.0, 10.0)	10.0 (9.0, 12.0)	**0.011**
Frailty (SOF scale)	144 (35.7%)	8 (17.0%)	136 (38.2%)	**0.004**
Falls	21 (5.2%)	1 (2.1%)	20 (5.6%)	0.491
Risk of undernutrition	30 (7.4%)	4 (8.5%)	26 (7.3%)	0.767

*ADL* activities of daily living, *IADL* instrumental activities of daily living, *CES-D* center for epidemiologic studies – Depression, *SOF* study of osteoporotic fractures index, *VI* visual impairment

^1^n (%); Median (IQR)

^2^Pearson’s Chi-squared test; Wilcoxon rank sum test; Fisher’s exact test

Table 2 shows the characteristics of participants with VI, according to gender and age categories. Among participants with VI, regardless of age group, there were significantly more women (than men) who lived alone, were professionally active, were overweight, with frailty syndrome and who were less likely to drive a car.

**Table 2:** Distribution of characteristics of people with visual impairment by gender in different age strata

Characteristic	Overall N = 356^1^	Under 65			Between 65 and 75			Over 75		
		Male N = 51^1^	Female N = 71^1^	p-value^2^	Male N = 89^1^	Female N = 95^1^	p-value^2^	Male N = 31^1^	Female N = 19^1^	p-value^2^
Age;median (IQR)	67.0 (63.0, 71.3)	61.0 (58.5, 63.0)	61.0 (59.0, 63.0)	> 0.9	68.0 (66.0, 71.0)	69.0 (66.0, 71.0)	0.7	77.0 (75.5, 79.0)	78.0 (76.0, 80.0)	0.3
Live alone	160 (44.9%)	5 (9.8%)	46 (64.8%)	** < 0.001**	9 (10.1%)	75 (78.9%)	** < 0.001**	6 (19.4%)	19 (100.0%)	** < 0.001**
Secondary education or higher	247 (69.4%)	43 (84.3%)	47 (66.2%)	**0.025**	66 (74.2%)	59 (62.1%)	0.080	23 (74.2%)	9 (47.4%)	0.055
Employed	141 (39.6%)	15 (29.4%)	44 (62.0%)	** < 0.001**	22 (24.7%)	47 (49.5%)	** < 0.001**	4 (12.9%)	9 (47.4%)	**0.018**
Able to drive	69 (19.4%)	17 (33.3%)	9 (12.7%)	**0.006**	27 (30.3%)	11 (11.6%)	**0.002**	5 (16.1%)	0 (0.0%)	NA
Overweight*	255 (71.6%)	39 (76.5%)	65 (91.5%)	**0.021**	52 (58.4%)	71 (74.7%)	**0.019**	14 (45.2%)	14 (73.7%)	**0.049**
Diabetes	37 (10.4%)	7 (13.7%)	3 (4.2%)	0.092	11 (12.4%)	12 (12.6%)	> 0.9	2 (6.5%)	2 (10.5%)	0.6
Hypertension	115 (32.3%)	13 (25.5%)	24 (33.8%)	0.3	21 (23.6%)	35 (36.8%)	0.051	0 (0.0%)	0 (0.0%)	NA
Stroke	9 (2.5%)	2 (3.9%)	1 (1.4%)	0.6	2 (2.2%)	4 (4.2%)	0.7			
Chronic alcoholism	26 (7.3%)	5 (9.8%)	2 (2.8%)	0.13	11 (12.4%)	4 (4.2%)	**0.044**	4 (12.9%)	0 (0.0%)	0.3
Tobacco consumption	6 (1.7%)	4 (7.8%)	0 (0.0%)	NA	2 (2.2%)	0 (0.0%)	NA	0 (0.0%)	0 (0.0%)	NA
Cognitive impairment	185 (52.0%)	17 (33.3%)	31 (43.7%)	0.2	42 (47.2%)	58 (61.1%)	0.059	19 (61.3%)	18 (94.7%)	**0.009**
ADL^§^	6.0 (6.0, 6.0)				6.0 (6.0, 6.0)	6.0 (6.0, 6.0)	0.6	6.0 (6.0, 6.0)	6.0 (6.0, 6.0)	**0.025**
IADL^§^	4.0 (4.0, 4.0)				4.0 (4.0, 4.0)	4.0 (4.0, 4.0)	0.4	4.0 (4.0, 4.0)	4.0 (3.0, 4.0)	**0.045**
Total CES-D	10.0 (9.0, 12.0)	10.0 (8.0, 12.0)	10.0 (8.0, 14.0)	0.3	10.0 (8.0, 12.0)	10.0 (10.0, 16.0)	**0.002**	10.0 (9.0, 12.0)	12.0 (10.5, 16.0)	**0.001**
Frailty (SOF scale)	136 (38.2%)	9 (17.6%)	28 (39.4%)	**0.010**	27 (30.3%)	43 (45.3%)	**0.037**	13 (41.9%)	16 (84.2%)	**0.003**
Falls	20 (5.6%)	0 (0.0%)	10 (14.1%)	NA	0 (0.0%)	4 (4.2%)	NA	1 (3.2%)	5 (26.3%)	**0.024**
Risk of undernutrition	26 (7.3%)	2 (3.9%)	1 (1.4%)	0.6	8 (9.0%)	10 (10.5%)	0.7	4 (12.9%)	1 (5.3%)	0.6
Appropriate correction of VI	275 (77.2%)	39 (76.5%)	61 (85.9%)	0.2	65 (73.0%)	76 (80.0%)	0.3	19 (61.3%)	15 (78.9%)	0.2

*ADL* activities of daily living, *IADL* instrumental activities of daily living, *CES-D* center for epidemiologic studies, score presented as median (Q1, Q3); Depression, *NA* not applicable, *SOF* study of osteoporotic fractures index, *VI* visual impairment

^1^n (%) * Overweight defined as Body mass index ≥ 25 kg/m^2^

^2^Wilcoxon rank sum test; Pearson’s Chi-squared test; Fisher’s exact test; Wilcoxon rank sum exact test

^§^There was no variation in this group, with values identical for all individuals (men and women). Scores presented as median (Q1, Q3)

Certain characteristics were distributed differently between men and women, depending on the age group considered. For example, level of education was significantly different only among those aged under 65, chronic alcoholism for those aged between 65 and 75, and cognitive decline, IADL and ADL scores and falls for those over 75 years. Among all these differences, the percentage of men was greater only for level of education and chronic alcoholism. Diabetes, hypertension, stroke, alcohol consumption, risk of undernutrition and having appropriate VI correction did not differ significantly between men and women, whatever the age group considered.

## Discussion

This study confirms the existence of age and gender differences in the health characteristics of older people with VI in Cameroon. Indeed, we found that for some characteristics, such as living alone, professional activity, overweight, frailty and driving a car, the sex difference was significant, whatever the age group. For others, such as level of education, chronic alcoholism, IADL and ADL scores and falls, the difference was only observed in certain age groups; while for the remaining variables, there were no differences in health characteristics between men and women.

The main limitation of our study was the self-reported nature of VI. However, this subjective measure is also commonly used in research and has been shown to be highly correlated with objective measures [18, 20].

The key strength of our study lies in addressing a significant knowledge gap, as very few studies have explored age and gender differences among visually impaired individuals in the general population of sub-Saharan Africa. Moreover, despite smaller numbers in certain groups, our total sample size provided aacceptable basis for meaningful comparisons across a wide range of characteristics, further reinforcing the value of our findings.

### Systematic differences

In sub-Saharan Africa, when studying differences between men and women, it is very important to take into account the socio-cultural context, especially when these differences are systematic regardless of age, as is the case for certain variables in our study. For example, the fact that women drive less or live alone more often is not necessarily linked to the context of their VI. Culturally, men drive more than women, and women often live more alone because of their longer life expectancy compared to men [21].

The socio-cultural context is not the only dimension to consider, notably when considering overweight. Women are generally more frequently overweight than men in sub-Saharan Africa and most other parts of the world [22]. The fact that more women than men are employed is also a reflection of society, although this could also be linked to VI. In fact, the vast majority of Cameroonians work in the informal sector [23], and women are more likely than men to do so. If VI was to lead to a reduction or cessation of activities, this would have a greater impact on those in the informal sector, who would find it harder to give up their source of income.

The difference in frailty between men and women in our study is consistent with the literature. Indeed, it has been shown that women are more vulnerable and frail than men. They live longer but are in poorer health, consistent with the male–female health-survival paradox [24, 25]. Biological, behavioral and social hypotheses have been put forward to explain this paradox [24]. For instance, estrogens have been shown to reduce the risk of death in females by postponing the onset of atherosclerosis, while testosterone has been shown to increase the risk of death in males by reducing the robustness of the male immunological system. However, it is important to note that pregnancy, childbirth and lactation may contribute to increased morbidity in females [24].

### Specific differences

With the exception of chronic alcoholism and education, all variables where differences between men and women appear only in certain age groups are considered in the literature to be consequences rather than causes of VI (like frailty). These include reduced autonomy (in terms of instrumental activities of daily living (IADL)), falls, risk of undernutrition and depression (as assessed by the CES-D) [26]. The differences between men and women cannot therefore be interpreted in the same way as others. Indeed, as VI is systematically more frequent in women than in men (see Table 1), it would be difficult to interpret these results in this work. However, we can point out that, like frailty, falls are probably significantly more prevalent in women than in men, but tests could not be carried out in the first two age groups due to zero numbers in men. These observations (also found elsewhere [27, 28]) may be explained by the fact that physically, men are more robust than women. For example, one study reported that women with VI showed significant associations between physical inactivity and depression and other mental health complications [27]. Another important fact to note in our results is that these differences between men and women mainly concern older people. In other words, as age advances, the differences between men and women intensify. This observation suggests a healthcare system that can rapidly target and care for younger women with VI, in order to limit the consequences of these impairments, which are more severe in women.

Chronic alcoholism is culturally stigmatized, as is tobacco use by women (although no differences were found). Women therefore tend to misreport in surveys, and this may be the reason why there were no reported cases of tobacco use by women in this study. This assumption about the stigma of chronic alcoholism among women is arguably less prevalent in contemporary times. Despite the absence of quantitative data, alcohol consumption among young women constitutes a significant public health concern in Cameroon. In an effort to address this issue, health authorities have implemented various awareness campaigns in the media. Given the method of recruitment in our study, it is possible that the participants are subjects who wish to take care of their health, which explains why there is only a small percentage of chronic alcoholism, making it impossible to detect differences across age groups.

### Differences between the visually impaired and the non-visually impaired

As shown in Table 1, there were some clear differences between people with and without VI. Our results are in line with the literature for most observations, notably physical frailty, which is clearly more present in people with VI [29–31]. Frailty is therefore a real marker to be on the lookout for in aging, particularly in people with VI. Like hypertension, diabetes was expected to be associated with VI [32, 33]. The lack of association in our study is probably linked to lack of statistical power.

### Positioning of our study in relation to WHO healthy aging objectives

In 2015, the WHO defined the goal of healthy aging in its world report as helping people to develop and maintain the functional capacities that enable well-being [3, 34]. In 2017, it published the integrated care for older people (ICOPE) guidelines aimed at improving, maintaining or limiting the decline in intrinsic abilities (locomotion; nutrition; vision; hearing; cognition; and psychology). These recommendations are evidence-based regarding appropriate approaches at the community level to detect and manage significant declines in physical and mental abilities, and to implement supportive interventions for caregivers [35]. Our study is in line with this approach, as it has identified groups at risk of a more rapid decline in intrinsic capacities, notably vision, locomotion (through physical frailty), cognition and depression with age. As women and older populations are most at risk, public health policies aimed at limiting or even eliminating early onset of decline in these populations are needed to align with the WHO's global goal of healthy ageing.

## Conclusion

There is a paucity of data reporting the characteristics of individuals with VI in sub-Saharan Africa. These initial descriptive results will enable health professionals and researchers to target the characteristics that merit particular attention, according to age and sex, in people with visual disorders. Age- and gender-specific differences show that VI disrupts the aging process, and early detection combined with appropriate follow-up (in accordance with the objectives of the ICOPE program, as promoted by the WHO) can limit the decline of those affected.

## Data Availability

No datasets were generated or analysed during the current study.
